# Carotid Endarterectomy With Stent Explantation for In-Stent Restenosis in Two Patients and Review of the Literature

**DOI:** 10.7759/cureus.101321

**Published:** 2026-01-11

**Authors:** Michael W Alchaer, Harrison Gorran, Anthony W Alchaer, Thomas A Abbruzzese

**Affiliations:** 1 General Surgery, HCA Healthcare/University of South Florida (USF) Morsani College of Medicine Graduate Medical Education (GME) HCA Florida Brandon Hospital, Brandon, USA; 2 Medicine, University of Kansas School of Medicine-Wichita, Wichita, USA

**Keywords:** carotid artery stenosis, carotid endarterectomy, in-stent restenosis, patch angioplasty, stent explantation

## Abstract

Carotid artery stenting (CAS) is a well-established alternative to carotid endarterectomy (CEA) for the treatment of carotid artery stenosis, particularly in patients at high surgical risk. However, in-stent restenosis (ISR) remains a notable long-term complication, occurring in up to 20% of patients within five years. The optimal management strategy for ISR remains unclear, as endovascular reintervention carries a significant risk of recurrence, and evidence supporting surgical explantation is limited.

We present two patients who developed severe carotid ISR and were successfully treated with CEA and complete stent explantation followed by bovine pericardial patch angioplasty. The first patient, an 84-year-old woman with bilateral carotid disease and a history of left carotid stenting, developed 90% restenosis at surveillance and underwent staged bilateral carotid revascularization with durable patency at three-year follow-up. The second patient, a 70-year-old man, presented with acute visual loss and imaging demonstrating near-occlusive thrombosis within a previously placed carotid stent. He underwent urgent surgical stent removal and patch angioplasty, achieving full recovery and maintaining patency at one year. Neither patient experienced perioperative neurological or cardiovascular complications.

These cases highlight the feasibility and safety of surgical stent explantation in the management of carotid ISR. Compared with repeat endovascular interventions, surgical removal directly addresses the underlying mechanical and neointimal causes of restenosis and may provide superior long-term durability in appropriately selected patients.

CEA with stent explantation represents an effective treatment option for ISR, particularly when endovascular options are limited by calcification, thrombus, or mechanical failure. Larger prospective studies are needed to establish patient selection criteria and optimize management strategies for this challenging clinical scenario.

## Introduction

Carotid artery stenosis remains a major contributor to ischemic stroke [1-3]. Historically, carotid endarterectomy (CEA) has been the gold standard, supported by randomized trials [1-3]. Over the past two decades, carotid artery stenting (CAS) has emerged as an alternative, particularly in high-risk surgical candidates [4]. However, with growing CAS utilization, in-stent restenosis (ISR) has become an increasingly recognized challenge [4,5].

ISR occurs in up to 20% of patients within five years of CAS, compared with approximately 9% after CEA [1-4]. Predictors include vessel size, calcification, and stent underexpansion [4,5]. Recent guideline updates from the European Society for Vascular Surgery emphasize that the optimal management of ISR remains undefined and should be individualized [6]. Randomized evidence further indicates that restenosis is more frequent after CAS than after CEA, reinforcing the rationale for careful patient selection and surveillance [1-3,7]. Recent cohort studies have also identified additional patient and procedural predictors for ISR [8,9].

While ISR has been reported more frequently in older patients and those with extensive atherosclerotic disease, current literature has not demonstrated a consistent sex-based predominance, and incidence appears largely driven by anatomic and procedural factors rather than demographic variables [4,5,8,9].

We present two patients with severe carotid ISR managed with CEA and complete stent explantation, representing both elective, surveillance-detected restenosis, and acute thrombotic ISR. These cases highlight the technical feasibility, clinical indications, and outcomes of surgical stent removal in scenarios where repeat endovascular intervention is unlikely to provide durable benefit, while contextualizing management within the existing literature [1-12].

## Case presentation

Surgical technique

All procedures were performed under general anesthesia with continuous electroencephalographic monitoring. A longitudinal incision was made along the anterior border of the sternocleidomastoid. After the division of the platysma, the internal jugular vein was mobilized, and the tributaries were ligated for exposure. The vagus nerve was identified and preserved.

The common, internal, and external carotid arteries, along with the superior thyroid, were dissected circumferentially and controlled with vessel loops. Following systemic heparinization, the arteries were clamped in sequence: internal, external, and then common.

An arteriotomy was initiated below the stent with a #11 blade and extended with Potts scissors. A precise endarterectomy plane was developed proximally and distally, allowing full stent removal along with neointimal hyperplasia. After achieving excellent proximal and distal endpoints, the arteriotomy was repaired with a bovine pericardial patch and 6-0 Prolene suture. Hemostasis was confirmed, and clamp removal was sequenced with back bleeding and flushing. Doppler confirmed the restored flow. The wound was closed in layers over a drain.

Case 1

The patient was an 84-year-old woman with a complex vascular history including bilateral high-grade carotid stenosis, hypertension, hyperlipidemia, and remote coronary artery disease. She was functionally independent and ambulatory, with no prior stroke or transient ischemic attack. She initially underwent left CAS several years earlier due to high surgical risk at that time, driven by unfavorable neck anatomy and medical comorbidities that made CEA less desirable.

Surveillance duplex ultrasound revealed a new 90% ISR of the left carotid artery in conjunction with severe contralateral carotid disease. Given her age, progression of disease, and presence of a previously stented vessel with neointimal hyperplasia, repeat endovascular intervention carried a high risk of further restenosis and mechanical failure. Therefore, open repair was favored. Multidisciplinary consensus concluded that CEA with full stent explantation offered the most durable treatment option, particularly given her life expectancy and preserved functional status.

**Figure 1:**
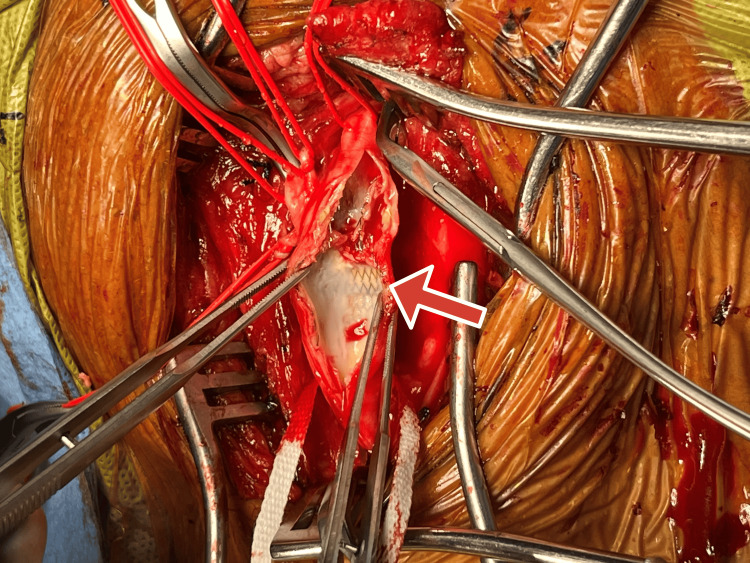
Intraoperative photograph demonstrating exposed carotid artery stent (arrow) during carotid endarterectomy prior to complete stent explantation Arrow pointing at stent previously placed. Dense neointimal hyperplasia surrounding the stent is visible

**Figure 2:**
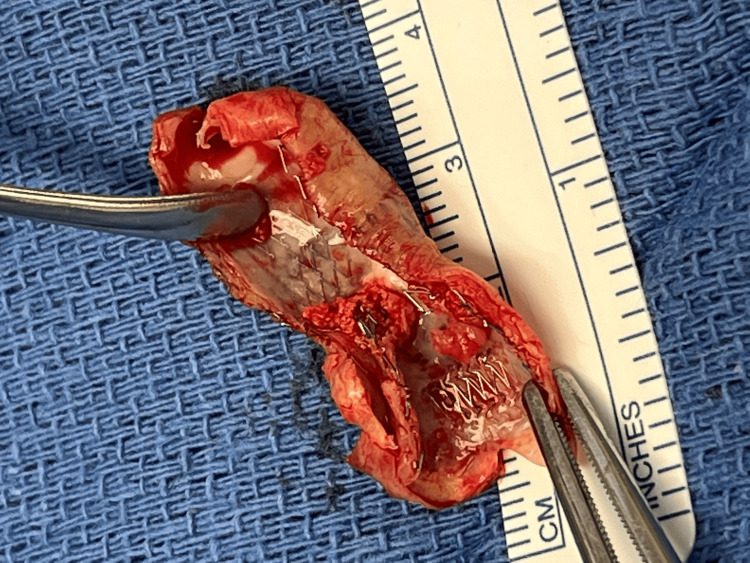
Explanted stent with adherent neointimal hyperplasia

**Figure 3:**
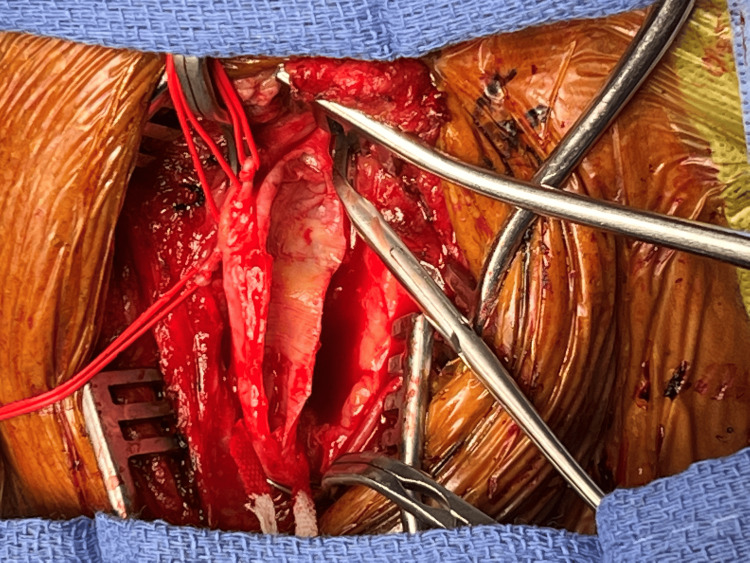
Intraoperative photo following patch angioplasty showing widely patent carotid bifurcation

She underwent staged repair right CEA for untreated severe stenosis, followed by left CEA with stent removal. This approach minimized perioperative stroke risk and ensured optimal cerebral perfusion. Her recovery was uncomplicated, and three-year surveillance confirmed continued patency (Figures 1-3).

Postoperative duplex ultrasound demonstrated widely patent carotid arteries without residual stenosis. Surveillance imaging at three years confirmed durable patency with no evidence of recurrent ISR or neurological events.

Case 2

The second patient was a 70-year-old man with a history of bilateral carotid stenting performed for symptomatic carotid stenosis several years prior. His comorbidities included diabetes, hypertension, and heavy arterial calcification on imaging. He was living independently and had no baseline neurological deficits prior to the acute presentation.

He presented with sudden left-eye visual loss, and imaging demonstrated a near-occlusive thrombus within his left carotid stent. The stent was noted to be externally compressed by dense circumferential calcification, an anatomic scenario in which repeat angioplasty or repeat stenting would be unlikely to achieve durable luminal gain and would carry significant thromboembolic risk.

Given the urgent ischemic presentation, rapid progression of thrombosis, and the mechanical nature of the failure, open surgery was deemed the safest definitive treatment. CEA with stent explantation allowed complete removal of thrombus, neointimal tissue, and the dysfunctional stent. He recovered completely and remained patent at one-year follow-up. 

Postoperative imaging confirmed restoration of luminal patency, and the patient remained neurologically intact. Duplex ultrasound at one-year follow-up demonstrated sustained patency without recurrent restenosis or thrombus.

## Discussion

ISR remains a major clinical challenge following CAS [1-5]. Rates as high as 20% at five years have been reported, with risk factors including small vessel diameter, heavy calcification, and incomplete stent expansion [3-5]. Although balloon angioplasty and repeat CAS are common treatment options, both are associated with significant recurrence rates and limited long-term durability [1,3,5]. CEA with stent explantation, though technically demanding, addresses the underlying pathology by removing both the stent and neointimal hyperplasia [5,10-12].

Multiple reports, including recent case series, have demonstrated favorable long-term patency after surgical explantation [10-12]. Reichmann et al. reported excellent one-year patency rates and no perioperative mortality following CEA for ISR, while Takahashi et al. and Stilo et al. also documented durable outcomes with minimal complications [5,10-12]. Our cases align with these findings, demonstrating feasibility and excellent results in both elective and urgent settings.

Recent guidelines and reviews acknowledge CEA with stent removal as an acceptable strategy when endovascular options are unsuitable [5,6,12]. Furthermore, randomized evidence indicates that restenosis is significantly more frequent after CAS than after CEA, reinforcing the rationale for definitive surgical correction [1-3]. Future research should focus on multicenter registries and comparative studies evaluating stent explantation versus repeat stenting to better define optimal management strategies for carotid ISR.

The two patients in this report represent distinct clinical scenarios. The first case involved ISR detected during routine post-CAS surveillance, consistent with guideline recommendations emphasizing close duplex monitoring because restenosis occurs more frequently after CAS than after CEA [1-3,6]. Early identification permitted an elective, staged approach before any neurological symptoms occurred.

In contrast, the second patient presented acutely with visual loss and near-occlusive thrombotic ISR. Acute thrombotic or mechanically compressed stents are situations where repeat endovascular intervention may be ineffective or unsafe, particularly in the presence of heavy calcification or stent underexpansion [4,5,8,9]. Recent reports have demonstrated that urgent CEA with stent explantation can be safely performed in such anatomically complex or unstable presentations [10-12]. Together, these cases highlight that CEA with stent removal is effective in both elective and emergent settings when appropriately selected.

These cases also raise the question of whether current post-CAS surveillance protocols are adequate. Multiple trials, including carotid revascularization endarterectomy vs stenting (CREST), endarterectomy vs angioplasty in patients with symptomatic severe carotid stenosis (EVA-3S), and International Carotid Stenting Study (ICSS), consistently show higher long-term restenosis rates following CAS compared with CEA [1-3,7]. Given that ISR can reach 20% at five years and is more common in patients with small vessels, heavy calcification, or incomplete stent expansion [3-5,8,9], certain high-risk patients may benefit from intensified or prolonged duplex ultrasound surveillance.

Our first case supports the value of structured monitoring to identify ISR before symptom onset, whereas the second case demonstrates that ISR can progress rapidly to acute ischemic events despite prior stenting. Further research is needed to determine which patient subgroups warrant enhanced surveillance and whether earlier detection could reduce the incidence of acute, symptomatic ISR.

## Conclusions

CEA with stent explantation represents a feasible and effective treatment option for carotid ISR, particularly in patients with heavy calcification, stent underexpansion, or mechanical stent failure in whom endovascular therapy is unlikely to succeed. Although technically demanding, existing case series and reports demonstrate excellent long-term patency and low recurrence rates when performed by experienced surgeons. These cases also highlight the importance of careful patient selection and structured post-CAS surveillance, given the higher long-term restenosis rates observed after CAS compared with CEA. Further research, including multicenter registries and comparative studies, is needed to refine selection criteria and establish evidence-based management strategies for this challenging clinical scenario.
